# Injury coding in a national trauma registry: a one-year validation audit in a level 1 trauma centre

**DOI:** 10.1186/s12873-019-0276-8

**Published:** 2019-10-30

**Authors:** Anna Bågenholm, Ina Lundberg, Bjørn Straume, Rune Sundset, Kristian Bartnes, Tor Ingebrigtsen, Trond Dehli

**Affiliations:** 1 Department of Radiology, University Hospital of North Norway, Sykehusveien 38 -, PO box 103, N-9038 Tromsø, Norway; 2 Department of Clinical Medicine, Faculty of Health Science, UiT-The Artic University of Norway, Tromsø, Norway; 3 Division of Cardiothoracic and Respiratory Medicine, University Hospital of North Norway, Tromsø, Norway; 4 Centre for quality improvement and development, University Hospital of North Norway, Tromsø, Norway; 5 Department of Community Medicine, Faculty of Health Science, UiT-The Artic University of Norway, Tromsø, Norway; 6 PET imaging Centre, University Hospital of North Norway, Tromsø, Norway; 7 Department of Neurosurgery, ENT and Ophthalmology, University Hospital of North Norway, Tromsø, Norway; 8 Department of Gastrointestinal Surgery, University Hospital of North Norway, Tromsø, Norway

**Keywords:** Trauma registry, Validation, Patient record, Audit, Abbreviated injury scale, Injury scoring

## Abstract

**Background:**

Hospitals must improve patient safety and quality continuously. Clinical quality registries can drive such improvement. Trauma registries code injuries according to the Abbreviated Injury Scale (AIS) and benchmark outcomes based on the Injury Severity Score (ISS) and New ISS (NISS). The primary aim of this study was to validate the injury codes and severities registered in a national trauma registry. Secondarily, we aimed to examine causes for missing and discordant codes, to guide improvement of registry data quality.

**Methods:**

We conducted an audit and established an expert coder group injury reference standard for patients met with trauma team activation in 2015 in a Level 1 trauma centre. Injuries were coded according to the AIS. The audit included review of all data in the electronic health records (EHR), and new interpretation of all images in the picture archiving system. Validated injury codes were compared with the codes registered in the registry. The expert coder group’s interpretations of reasons for discrepancies were categorised and registered. Inter-rater agreement between registry data and the reference standard was tested with Bland–Altman analysis.

**Results:**

We validated injury data from 144 patients (male sex 79.2%) with median age 31 (inter quartile range 19–49) years. The total number of registered AIS codes was 582 in the registry and 766 in the reference standard. All injuries were concordantly coded in 62 (43.1%) patients. Most non-registered codes (*n* = 166 in 71 (49.3%) patients) were AIS 1, and information in the EHR overlooked by registrars was the dominating cause. Discordant coding of head injuries and extremity fractures were the most common causes for 157 discordant AIS codes in 74 (51.4%) patients. Median ISS (9) and NISS (12) for the total population did not differ between the registry and the reference standard.

**Conclusions:**

Concordance between the codes registered in the trauma registry and the reference standard was moderate, influencing individual patients’ injury codes validity and ISS/NISS reliability. Nevertheless, aggregated median group ISS/NISS reliability was acceptable.

## Background

Faced with increasing pressure to reduce costs, hospitals must minimize waste through continuous improvement of patient safety and quality. Timely provision of process and outcome data from clinical quality registries to clinicians has been shown to drive such improvements in healthcare [1–4]. In 1976, the American College of Surgeons Committee on Trauma introduced the trauma registry as part of the trauma system [5]. Injury description and grading of injury severity are systematically registered [6–8]. This provides benchmarking data for comparisons of quality of care between patients and institutions, and facilitates continuous improvement [1, 9]. Norway introduced a national trauma system in 2007 [10] and the national trauma registry (NTR) was established in 2015 [11].

Many studies on validation of the Abbreviated Injury Scale (AIS) injury coding have been published [12–14]. They typically report inter-rater variability between trauma registry coders based on samples where several AIS-coders code the same patient, and generally show low inter-rater agreement between coders for actual AIS codes. Such studies do not, however, validate the injury data quality in the trauma registry itself. Few report validation of injury codes in trauma registries. Horton et al. [15] compared the initial registration in a registry with a second blinded re-registration by an AIS certified audit coder, and found satisfactory inter-rater agreements on the number of AIS codes. A more comprehensive approach is to establish a reference standard by using an expert coder group to review all information in the patient record and recode all injuries. To our knowledge, this has not been done for trauma registries. The University Hospital of North Norway Tromsø campus (UNN) is the Level 1 trauma centre for northern Norway and started registration in the NTR 01.01.2015. This is a validation study of the injury coding quality during the first year. We compare a consensus coding by an expert coder group to the routine NTR data entry. The primary aim was to validate the injury codes and severities registered in the trauma registry. Secondarily, we aimed to examine causes for missing and discordant codes, to guide improvement of registry data quality.

## Methods

### Study type, population and region

This is a clinical audit. An expert coder group validated injury codes and compared them to the routine injury code input in a trauma registry. Trauma registry coders continuously survey lists of emergency admissions and prospectively register all trauma patients fulfilling predefined criteria in the NTR. In this study, we included all patients admitted with trauma team activation (TTA) in 2015, registered in NTR at UNN. Criteria for TTA include vital functions, extent and mechanism of injury, and have been described previously [16]. The UNN trauma centre covers a population of 486,792 spread over a rural area of 257,000 km^2^ (1.9 inhabitants per km^2^) [17, 18]. It supports ten referring hospitals. Study data entry continued until death, or discharge home or to rehabilitation.

### Injury coding

The registry codes injuries according to the AIS code manual [6, 19]. The AIS classifies injuries with a six-digit anatomical code, and adds a severity score ranked from one (injuries minimal in severity, such as subcutaneous hematomas) to six (injuries maximal in severity, currently untreatable). Only certified AIS coders have access to the manual [6]. Coders manually assign all injuries an AIS code, and the registry automatically calculates the Injury Severity Score (ISS) and the New ISS (NISS). Baker et al. introduced the ISS in 1974 after showing that summarizing the square of the highest AIS score in three of six body regions shows a good correlation to survival [7]. Patients with an ISS > 15 are defined as severely injured. The same group introduced the NISS in 1997 [8]. The NISS is the summation of the square of the three highest AIS score injuries, regardless of body region. NISS is easier to calculate and predicts survival better than the ISS [8]. Three coders certified in the AIS 2005 Update 2008 manual [6] did the injury coding according to the AIS convention. They had 10% coding employments and no clinical role. Coding was performed after patient death or hospital discharge. They were two medical students with two (IL) and 3 years coding experience, and one nurse with 6 month coding experience. They used pre- and intra-hospital electronic health records (EHR) including the radiology information system (RIS) to identify and code all injuries.

### Reference standard

The expert group consisted of the first (AB) and second (IL) authors. AB is a AIS certified coder and a senior radiologist with 10 years of experience in trauma care. IL is a AIS certified junior medical doctor with experience as trauma coder since 2014. AB made a blinded new AIS injury assessment of all study patients between February 29 and July 31 2016. This included review of the EHR, and new interpretations of all diagnostic imaging in the picture archiving and communication system (PACS). The new interpretation was compared to the RIS report to identify all codes missing in the original registry coding due to incomplete radiology reports. Injury codes were set using the AIS 2005 Update 2008 manual. ISS and NISS were calculated manually, and all study data were registered in a Microsoft Excel spreadsheet. Next, IL retrieved AIS codes, ISS, and NISS from the NTR, and the data were entered into the same spreadsheet during the autumn of 2016. Finally, AB and IL made an expert coder group consensus coding on all patients during January through Mars 2017, and thereby established a reference standard. In cases of complete agreement between AIS codes, this was verified. In cases of discrepancies between a registry code and the new assessed AIS code, a consensus code was set. This included a second reassessment of diagnostic imaging in cases of discrepancies between the new radiological interpretations and the RIS reports. When appropriate, the expert coder group discussed cases with other senior radiologists or other specialists. When in doubt about a correct understanding of the AIS coding manual, they consulted a senior AIS code instructor at the largest trauma centre in Norway. Causes for missing and discordant AIS codes in the registry were categorised as related to the patient record, radiology report, AIS manual or as other causes. Discordant AIS codes were categorised as either coding of a non-existent injury, or discordant AIS code with concordant or discordant severity grade. To assess the overall completeness of AIS coding per patient, we divided the concordant number of AIS codes in the registry by the total number of reference standard codes. According to the AIS manual, all injuries, including subcutaneous hematomas, shall be coded separately, even when multiple AIS severity 1-codes do not influence ISS. We report overall completeness with and without correction for more than one missing multiple AIS 1-code [14].

### Statistics

Statistical analysis was performed with IBM SPSS Statistics 23. Descriptive and frequency statistics were used and normality tested with histograms, Kolmogorov–Smirnov and Shapiro–Wilk tests. Abnormally distributed data are presented as medians with 25 and 75 inter-quartile range (IQR).

A Bland–Altman analysis was used to report agreement for ISS and NISS in the registry compared to the reference standard. We plotted the mean between the paired measured ISS in a Bland–Altman plot, calculated for each patient by summarizing the ISS in the trauma registry and the reference standard, and dividing by two on the X-axis. The Y-axis shows the difference between the paired ISS, calculated as ISS in the trauma registry subtracted the reference standard ISS. With ideal agreement the difference equals zero [20, 21]. NISS was plotted in the same way. This method requires normality distribution of the difference variable [22]. In the regression analysis, *p* values < 0.05 were considered significant.

## Results

### Descriptive analysis of the population

Table 1 shows characteristics of the 144 patients in the study population. The ten patients, who died within 30 days after trauma, had an ISS range 22–45.

**Table 1:** Characteristics of the trauma population (*n* = 144)

Characteristics	
Male sex, n (%)	114 (79.2)
Age, years in median (IQR)	31 (19–49)
Age groups, n (%)	
0–16	26 (18.1)
> 16	118 (81.9)
Trauma mechanism	
Penetrating traumas, n (%)	5 (3.5)
Blunt, n (%)	139 (96.5)
Cause of incident, n (%)	
Road traffic	63 (45.3)
Snowmobile	11 (7.9)
Fall	31 (22.3)
Hit by blunt object	13 (9.3)
Explosion/fire	8 (5.7)
Avalanches and/or hypothermia	8 (5.8)
Other causes	5 (3.6)
Transferred from other hospitals, n (%)	36 (25.0)
Length of stay, median days (IQR)	4 (1.2–11.5)
30-day mortality, n (%)	10 (6.9)
Head injuries	6 (4.2)
Other causes	4 (2.8)

*IQR* Inter-quartile range.

### Quality of registered AIS codes

The total number of registered AIS codes in the 144 patients was 582 in the registry and 766 in the reference standard.

The total number of missing and discordant AIS codes in the registry was 369. In 17 patients, we found 46 missing codes, all identical with another AIS code recorded in the same patient. The data retrieval from the NTR returned only one of these identical codes. After correction for this error, a total of 323 missing and discordant codes remained for analysis. Table 2 shows the results from division of the concordant number of AIS codes in the registry by the total number in the reference standard per patient. More than 75% agreement was reached for 47.2% of the patients. Subtracting the minor external lacking AIS 1 injuries not affecting ISS (*n* = 94) increased the proportion to 62.5%.

**Table 2:** Quality of concordant AIS codes in UNN Trauma registry

Concordant number of AIS codes in UNN trauma registry divided with the total number of expert group codes per patient	Original AIS data output from the Norwegian national trauma registry		Original data output adjusted for minor external missing injuries not affecting injury severity	
	Frequency n (%)	Cumulative %	Frequency n (%)	Cumulative %
100% concordant	47 (32.6)	32.6	62 (43.1)	43.1
99–75% concordant	21 (14.6)	47.2	28 (19.4)	62.5
74–50% concordant	43 (29.9)	77.1	35 (24.3)	86.8
49–25% concordant	17 (11.8)	88.9	10 (6.9)	93.8
24–0% concordant	16 (11.1)	100.0	9 (6.3)	100.0

*AIS* Abbreviated Injury Scale, *UNN* University Hospital of North Norway

### Missing AIS codes

In total, 212 missing AIS codes were found in 75 (52.1%) of the 144 patients (range 1–14 missing codes per patient). After correcting for the 46 codes not included in data retrieval from the NTR, 166 missing codes in 71(49.3%) patients (range 1–10 missing codes per patient) remained for analysis.

Table 3 shows the causes for the 166 missing codes. We analysed on the level of each patient and registered the missing codes into the cause-categories. Each cause was counted only one time for each patient. Information in the EHR overlooked by the coders was the dominating cause. Most overlooked injuries were minor (AIS 1). Examples are hematomas only described in nurse reports or injuries identified on radiology examinations described in the RIS only. Also, three injuries described as suspected in the RIS, not coded in the registry in accordance with the AIS manual, were concluded to be injuries in the reference standard.

**Table 3:** Causes for missing and discordant AIS codes in the UNN trauma registry 2015

	Missing AIS code		Discordant AIS code			
	AIS ≥ 2 ^b^ injury grades	AIS < 2 ^b^ injury grades	Injury not existing	AIS ^b^ injury grade discordant	AIS ^b^ injury grade concordant	
Decided audit cause ^a^						Total
Related to the patient record						
Trauma registrar overlooked information	22	42				64
Trauma registrar misinterpreted information ^c^	6	3	9	0	0	18
Trauma registrar chose incorrect AIS code ^d^			0	26	22	48
Trauma registrar got information difficult to interpret			0	2	1	3
Trauma registrar used radiological DAI criteria ^e^			0	2	0	2
Trauma registrar used NFS code instead of a more specified code			0	2	14	16
Trauma registrar coded injury but other AIS code chosen included the injury			6	0	0	6
Trauma registrar double coded injury by mistake			2	0	0	2
Related to the radiology report						
Injuries not described	4	8				12
Injuries inaccurate described	3	0	7	8	12	30
Related to the AIS manual						
AIS guide lacks code for cardiac arrest due to hypothermia			2	0	0	2
Related to other reasons						
Physician described fracture not existing, radiology report correct			1	0	0	1

*AIS* Abbreviated Injury Scale, *UNN* University Hospital of North Norway, *DAI* diffuse axonal injury, *NFS* Not further specified, ^a^ Analysed on the level of each patient, each cause was counted only one time for each patient, ^b^ AIS Injury grade severity ranking 1–6, ^c^ Misinterpreted information corresponds to patient record information understood incorrectly, ^d^ Correct understanding of information but an incorrectly chosen code, for example, a mix of intracerebral contusion bleeding AIS code with the brain contusion code, ^e^ DAI criteria for radiological description do not fully comply with the DAI criteria in the AIS code manual

### Discordant AIS codes

Table 3 also shows the 157 discordant AIS codes registered in 74 (51.4%) of the 144 patients (range 1–9 discordant codes per patient). We analysed on the level of each patient and registered the discordant codes into the cause-categories. Each cause was counted only one time for each patient. Discordant coding and injury grading of existing injuries were most common, followed by use of an unspecified code for injuries that could have been coded with a specific code.

Table 4 shows an overview of the 157 discordantly coded injuries. Discordant coding of head injuries and extremity fractures were most frequent.

**Table 4:** Description of the 157 injuries with discordant AIS codes in the trauma registry

Type of injury	Discordant AIS code for a injury not existing	Discordant AIS code with discordant AIS injury grade ^a^	Discordant AIS code with concordant AIS injury grade ^a^	
Head/face/spine				Total
Spinal and cranial fracture	0	12	11	23
Face fracture	0	2	4	6
Intracranial parenchymal haemorrhage	0	6	9	15
Intracranial subarachnoid haemorrhage	0	5	0	5
Intracranial epi/subdural haemorrhage	0	1	1	2
Diffuse axonal injury	0	3	1	4
Cerebral concussion	0	3	0	3
Thorax				
Lung contusion	2	2	0	4
Pneumothorax	0	6	0	6
Costa fracture	0	6	3	9
Abdominal				
Thoracoabdominal injury	0	5	0	5
Extremity				
Fracture/joint dislocation	14	3	33	50
External and other reasons				
External (hematoma, laceration, burn injury)	2	4	9	15
Hypothermia	1	0	0	1
Other reason	9	0	0	9
Total	28	58	71	157

*AIS* Abbreviated Injury Scale, ^a^ AIS Injury grade severity ranking 1–6.

### Agreement between ISS/NISS

For the total population, ISS and NISS were positively skewed towards less severe injuries (mode ISS 1) both in the registry and the reference standard. Median ISS score was 9 in both data sets (range 0–75 and IQR 2–17 in the registry, range 0–59 and IQR 2–22 in the reference standard). Median NISS score was 12 in both data sets (range 0–75 and IQR 2–27 in the registry, range 0–66 and IQR 3–27 in the reference standard). After exclusion of the eight uninjured patients (ISS score 0), median ISS score was 9 (range 1–75, IQR 4–19) in the registry. After exclusion of the six patients with ISS 0 in the reference standard median ISS was 10 (range 1–59, IQR 4–22). Median NISS score remained 12 in both data sets (range 1–75, IQR 4–27 in the registry and range 1–66, IQR 4–27 in the reference standard) after exclusion of the uninjured patients.

In the reference standard, 52 (36.1%) patients had an ISS > 15, and 64 (44.4%) a NISS > 15. Fifty-two (36.1%) had a change in ISS from the registry to the reference standard. Six (4.2%) with ISS ≤15 in the registry got an ISS > 15, and two (1.4%) with ISS > 15 in the registry got an ISS ≤15. Fifty-eight incorrect AIS codes among 40 patients in the registry had a discordantly chosen injury grade. Thirty-eight had injuries which severity were graded to low. AIS 2 changed to 3 were most common (16 changes). Twenty patients had injuries which severity were graded to high. AIS 3 changed to 2 was most common (6 changes).

**Fig. 1:**
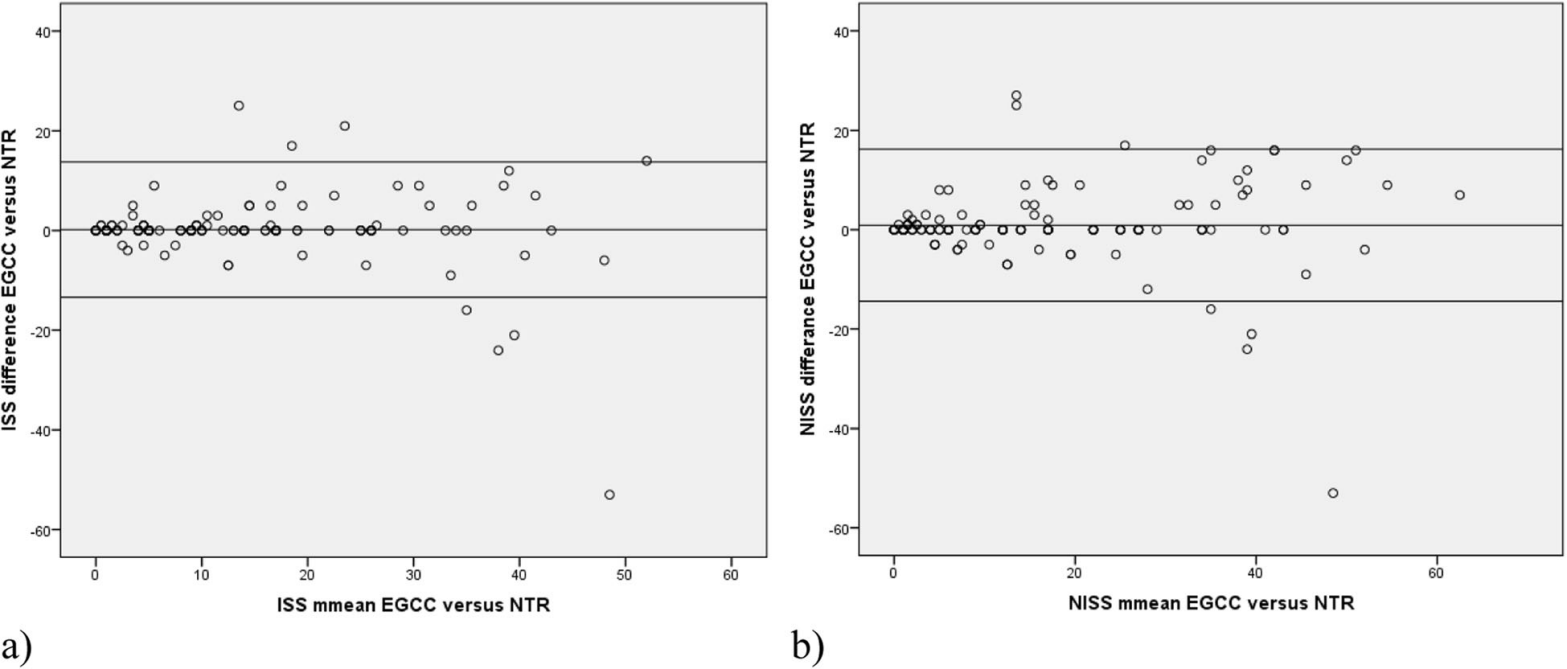
Bland–Altman plot for ISS and NISS in NTR versus the reference standard. Bland–Altman ISS and NISS plot for 144 patients quantify agreement in the national trauma registry (NTR) compared to the expert group consensus coding (EGCC). The X -axis presents the mean between the paired measured (**a**) ISS and (**b**) NISS in the EGCC and the NTR. Y-axis presents the difference between the paired a) ISS b) NISS in the EGCC versus the NTR. Mean difference ISS 0.194, 95% CI (± 2SD of the mean difference) upper limit + 13.8 and lower limit − 13.4. Mean difference NISS 0.924, 95% CI (± 2SD of the mean difference) upper limit + 16.2, and lower limit–14.4

Histograms (not presented) of differences between the trauma registry and the reference standard ISS and NISS, approximated normal distribution. Figure 1 shows Bland–Altman scatter-plots of the mean (x-axis) between the paired measure of ISS (a) and NISS (b) in the trauma registry and the reference standard versus the difference between them (y-axis). The plots show no proportional bias. Regression analysis showed no significant differences neither for ISS (*p* = 0.078) or NISS (*p* = 0.656). The outlier in the plot represents one patient registered with an AIS 6 crush injury code, scoring the patient to ISS 75, while the reference standard set ISS to 22 due to the lack of diagnostics, autopsy or surgery, according to the AIS manual.

## Discussion

The main finding in this validation study is that complete coding in a trauma registry is challenging to achieve, even with AIS certified and trained coders. Full concordance between the original coding in the trauma registry and the reference standard occurred in 43.1% of the patients. Most of the observed disagreement was at the lower injury severity. The most common causes for missing or discordant codes were that coders overlooked information in the EHR, or assigned discordant AIS codes. This caused a discordant ISS in 53 (36.8%) patients. It did not, however, influence median ISS or NISS for the total population in the registry, as the median scores were the same for ISS (9) and NISS (12) in the registry and the reference standard.

### AIS coding quality

Horton et al. [15] studied a randomly selected sample of 450 patients from the Dutch national trauma registry. They compared the registered number of AIS codes with the number in a second, blinded re-registration by an experienced audit coder, and found agreement in 63% of cases. The causes for disagreement and the frequency of discordant codes were not studied. Ringdal et al. [14] studied inter-rater agreement in a representative group of Norwegian trauma registry coders, and compared with a reference standard set by a panel of AIS coding experts. Fifty patient cases were selected from the registry at Oslo University Hospital. Overall, 61.5% of the AIS codes assigned by the coders agreed with the reference standard, but comparison with the codes originally entered into the registry was not done. Neale et al. [13] also studied inter-rater agreement between registry coders. They randomly selected 120 cases from the Queensland trauma registry for re-coding, and found that on average, 39% of the codes used by any two coders for each of the injured persons were identical. Again, comparison with the original registry data was not done. Summarised, the inter-rater agreement between coders, and between coders and reference standards generally is low.

To our knowledge, the present study is the first to compare all injury codes in a registry population with a reference standard. Agreement between registry AIS codes and the reference standard was moderate. Accordingly, validation of data quality is necessary when individual level registry injury codes are used for quality improvement or research purposes [2].

The most common causes for missing or discordant AIS codes were information in the EHR overlooked by the coders. We consider incomplete summaries of the available information in physicians’ notes as the most likely underlying root cause. This could be more common among trauma patients as many clinicians from different specialties often share responsibility. In comparison, discordant radiological descriptions were a minor problem. Routine audit by trauma responsible senior clinicians could improve injury coding quality, but is resource demanding [23]. Instead, we have trained and certified trauma care physicians in AIS coding to improve their skills in describing injuries in the EHR. We anticipate this will facilitate communications between physicians and coders, and thereby improve the coding. Further, we now suggest coding review is included in our monthly trauma audit.

Two coding problems related to the AIS code manual were identified by the expert group. First, radiological criteria routinely used to diagnose diffuse axonal injuries and brain contusion do not fully comply with the AIS manual. This caused incorrect coding, and coder education and better code instructions could improve this. Second, two patients with hypothermic cardiac arrest were incorrectly coded as asphyxia cardiac arrest. Hilmo et al. [24] reported only 9 (26%) survivors among 34 patients with hypothermic cardiac arrest. This suggest an ISS of 50 as more accurate than the score of 25 [7] this patient group receives following the present AIS manual, lacking a hypothermic cardiac arrest code. We suggest that a specific code for hypothermic cardiac arrest should be added to the AIS code manual.

Our study revealed a software error causing under-reporting of injuries in data retrieved from the NTR. The error has been corrected by the registry administration. Unnoticed registry code retrieval problems may exist in other registries as well. This highlights the importance of early validation studies of new quality registries [25, 26].

### ISS and NISS scores

In some patients, different AIS codes in the trauma registry and the reference standard did not influence the ISS, but discordant AIS coding can influence prediction of mortality risk. This is a known problem with ISS and NISS. Different AIS triplets with the same ISS have different mortality [27]. Blunt and penetrating traumas with the same AIS values also show different mortality [28].

Interestingly, suboptimal AIS code quality in the registry did not influence population median ISS and NISS. This is in accordance with previous studies of AIS coding inter-rater variability and ISS/NISS [13–15, 23]. Accordingly, comparison of median ISS and NISS between institutions might be acceptable without correction of AIS codes in the trauma registry, allowing benchmarking across institutions. We advocate validation of this finding in a multicentre trauma registry study, as confirmation of this finding would improve trust in such benchmarking across institutions using routine trauma registry AIS codes.

### Limitations and strengths

Our study sample is relatively small, because the study was done as a quality audit of our data entry during the first year of registration in the NTR. Power analysis with sample size calculation was not done. This is a limitation. Results may not be generalizable, as different registries have different patient profiles and different injury pattern. Also, 57 patients registered in the trauma registry without TTA were not included. This entails a risk for selection bias, but we find it unlikely that inclusion of these less severely injured patients would have changed the impression of our overall injury coding quality. Further, one expert coder (AB) countersigned 27 trauma CT examination reports written by residents. The other expert coder (IL) participated in the original data registration in the registry by coding 81 (56%) of the patients. Thus, a risk for recall bias during establishment of the reference standard is present, but we consider it unlikely that this has influenced the results significantly. A bias caused by propensity to miscode particular injuries could also exist. However, a sensitivity analysis (not presented) in which we compare the analysis presented in Table 2 stratified by coders showed no such tendency. Further, in case of discrepancy, a risk for bias towards systematically weighting one of the expert coders more than the other could exist. This was counteracted by consulting other specialists in most cases of disagreement.

The major strength, compared to previous studies, is the rigorous validation through establishment of a reference standard for comparison with registry codes.

## Conclusions

Concordance between the codes registered in the trauma registry and the reference standard was moderate, influencing individual patients’ injury codes validity and ISS/NISS reliability. Nevertheless, aggregated median group ISS/NISS reliability was acceptable.

## Data Availability

Some parts of the data that supports the findings of this study are available from the corresponding author upon request, but most of the data are due to the form of a clinical audit not available. Important data are included in the article.

## Abbreviations

AIS: Abbreviated Injury Scale
EHR: Electronic health records
IQR: Inter-quartile range
ISS: Injury Severity Score
NISS: New Injury Severity Score
NTR: National Trauma Register
RIS: Radiology Information System PACS Picture Archiving and Communication System
TTA: Trauma team activation
UNN: University Hospital of North Norway
